# Role of APP Interactions with Heterotrimeric G Proteins: Physiological Functions and Pathological Consequences

**DOI:** 10.3389/fnmol.2017.00003

**Published:** 2017-01-31

**Authors:** Philip F. Copenhaver, Donat Kögel

**Affiliations:** ^1^Department of Cell, Developmental and Cancer Biology, Oregon Health & Sciences University, PortlandOR, USA; ^2^Experimental Neurosurgery, Goethe University FrankfurtFrankfurt am Main, Germany

**Keywords:** Alzheimer’s disease, amyloid precursor protein, APPL, *Drosophila*, Gαo, *Manduca*, migration, stress signaling

## Abstract

Following the discovery that the amyloid precursor protein (APP) is the source of β-amyloid peptides (Aβ) that accumulate in Alzheimer’s disease (AD), structural analyses suggested that the holoprotein resembles a transmembrane receptor. Initial studies using reconstituted membranes demonstrated that APP can directly interact with the heterotrimeric G protein Gαo (but not other G proteins) via an evolutionarily G protein-binding motif in its cytoplasmic domain. Subsequent investigations in cell culture showed that antibodies against the extracellular domain of APP could stimulate Gαo activity, presumably mimicking endogenous APP ligands. In addition, chronically activating wild type APP or overexpressing mutant APP isoforms linked with familial AD could provoke Go-dependent neurotoxic responses, while biochemical assays using human brain samples suggested that the endogenous APP-Go interactions are perturbed in AD patients. More recently, several G protein-dependent pathways have been implicated in the physiological roles of APP, coupled with evidence that APP interacts both physically and functionally with Gαo in a variety of contexts. Work in insect models has demonstrated that the APP ortholog APPL directly interacts with Gαo in motile neurons, whereby APPL-Gαo signaling regulates the response of migratory neurons to ligands encountered in the developing nervous system. Concurrent studies using cultured mammalian neurons and organotypic hippocampal slice preparations have shown that APP signaling transduces the neuroprotective effects of soluble sAPPα fragments via modulation of the PI3K/Akt pathway, providing a mechanism for integrating the stress and survival responses regulated by APP. Notably, this effect was also inhibited by pertussis toxin, indicating an essential role for Gαo/i proteins. Unexpectedly, C-terminal fragments (CTFs) derived from APP have also been found to interact with Gαs, whereby CTF-Gαs signaling can promote neurite outgrowth via adenylyl cyclase/PKA-dependent pathways. These reports offer the intriguing perspective that G protein switching might modulate APP-dependent responses in a context-dependent manner. In this review, we provide an up-to-date perspective on the model that APP plays a variety of roles as an atypical G protein-coupled receptor in both the developing and adult nervous system, and we discuss the hypothesis that disruption of these normal functions might contribute to the progressive neuropathologies that typify AD.

## APP As An Unconventional G Protein-Coupled Receptor: Historical Perspective

**FIGURE 1:**
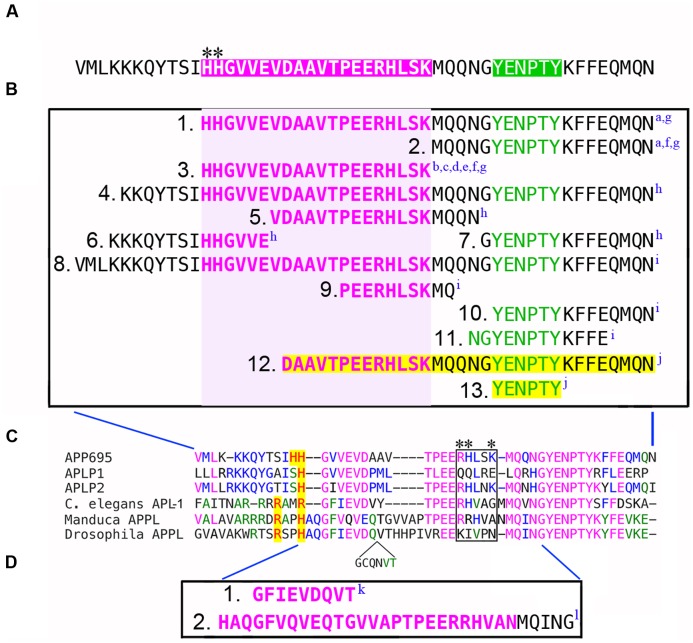
**Defining the G protein-binding domains in APP family proteins.** **(A)** Intracellular domain of human APP_695_, equivalent to the cytoplasmic AICD fragment generated by γ-secretase processing. Magenta region indicates the “peptide 20” Go binding domain (H_657_-L_676_) originally identified by Nishimoto et al. (1993); green region indicates the tyrosine-based sorting motif (YENPTY) that mediates interactions with many other cytosolic proteins. Asterisks indicate the N-terminal HH doublet in the Go domain, while the BBXXB motif (RHLSK) is located at the C-terminus (compare with **C**). **(B)** Summary of the deletions used by different investigators to map the sequences in APP that are required for its interaction with Gαo. Amino acids contained within the G protein-binding domain are highlighted in magenta; the YENPTY domain is highlighted in green. Deletions that eliminated Gαo interactions (1B_1_, 1B_3_, 1B_4_, 1B_5_, 1B_6_, 1B_8_, 1B_9_) encompassed some or all of the Go domain (indicated by light magenta box). Deletions that encompassed the YENPTY domain but not the Go domain did not affect Gαo interactions (1B_2_, 1B_7_, 1B_10_, 1B_11_). In one study, deletions highlighted in yellow (1B_12_, 1B_13_) were found to interfere with APP-Gαo signaling but paradoxically not with APP-Gαo interactions. Superscripted letters indicate citations for each deletion construct (summarized below). **(C)** Amino acid alignment of the G protein-binding domains from human APP_695_, APLP1 and APLP2; plus APL-1 from *Caenorhabditis elegans*, APPL from *Manduca sexta*, and APPL from *Drosophila melanogaster* (which contains an additional inserted sequence; shown below the alignment). Identical amino acids are indicated by color. Basic amino acids in that align with (or near) the HH doublet in APP_695_ are highlighted in yellow. The boxed region indicates the BBXXB motif in APP_695_ (RHLSK), and the equivalent region in other APP family proteins; only APLP2 also has a complete BBXXB motif (RHLNK). Asterisks indicate amino acids within the G protein-binding domains of APP_695_ and APLP1 that were found to be necessary for interactions between membrane-tethered AICDs or CTF fragments of the holoproteins and Gαs (Deyts et al., 2012). **(D)** Deletions in APPL that interfere with Gαo-associated motile responses in developing neurons (1D_1_, 1D_2_) and prevent direct binding between APPL and Gαo (1D_2_). Citations describing each deletion construct are as follows: ^a^Nishimoto et al., 1993; ^b^Okamoto et al., 1996; ^c^Ikezu et al., 1996; ^d^Yamatsuji et al., 1996a; ^e^Yamatsuji et al., 1996b; ^f^Hashimoto et al., 2000; ^g^Sudo et al., 2001; ^h^Sola Vigo et al., 2009; ^i^Milosch et al., 2014; ^j^Shaked et al., 2009; ^k^Torroja et al., 1999b; ^l^Ramaker et al., 2013.

Members of the APP family share many of the structural features that distinguish type-1 transmembrane receptors, including evolutionarily conserved extracellular domains capable of binding a variety of candidate ligands, plus highly conserved intracellular domains that can mediate interactions with numerous cytoplasmic adapter and signaling proteins (Turner et al., 2003; Jacobsen and Iverfeldt, 2009; Deyts et al., 2016b). In addition, APP is also capable of both homodimeric binding (to itself) and heterodimeric interactions with two APP-like proteins (APLP1 and APLP2) and other membrane-associated proteins (Scheuermann et al., 2001; Soba et al., 2005; Wang et al., 2009; Kaden et al., 2012), consistent with the perspective that APP and its orthologs can function as neuronal receptors that modulate both physiological and pathological responses. Whereas receptors with the topology of APP are most commonly associated with the activation of intracellular kinases (Heldin et al., 2016; Trenker et al., 2016), a growing number of single-pass receptors have now been shown to function as authentic G protein-coupled receptors (GPCRs) that mediate cellular responses via heterotrimeric G proteins, including Fibroblast Growth Factor and Epidermal Growth Factor Receptors (Patel, 2004; Hawkes et al., 2007). Based on the identification of a short motif in Insulin-like Growth Factor II receptor that binds the heterotrimeric G protein Gi (Okamoto et al., 1990), Nishimoto et al. (1993) identified a similar motif in APP (**Figure 1A**; described below), suggesting that APP might also function as G protein-interacting receptor. Specifically, they identified a 20 amino acid peptide (“peptide 20”) within the intracellular domain (His_657_-Lys_676_; numbering in APP_695_) that could directly bind and activate heterotrimeric G proteins containing Gαo but not other Gα subunits (including Gαs, Gαi_1_, Gαi_2_, and Gαi_3_) in reconstituted membranes (**Table 1**). This effect was blocked by PTX (a selective inhibitor of the Gαo/i subfamily). They also demonstrated that the alpha subunit of Go (Gαo) but not Gαi could be co-immunoprecipitated with APP from concentrated brain membranes, an interaction that was inhibited by adding excess peptide 20. Using membrane preparations from transfected SF9 cells, they then showed that APP_695_ could be co-immunoprecipitated with purified bovine Go, in contrast to mutated forms of APP lacking the peptide 20 domain (Nishimoto et al., 1993). Of note is that Gβ could also be detected in these immunoprecipitates, consistent with the model that APP normally interacts with Go as a heterotrimeric complex (similar to conventional GPCRs). Lastly, Gαo was shown to specifically mediate the effects of peptide 20 on GTP hydrolysis, while pre-treatment with GTPγS blocked this interaction (Lang et al., 1995), indicating that the activation state of Go regulates its interaction with APP (again consistent with conventional GPCRs).

**Table 1:** Evidence for functional interactions between APP family proteins and heterotrimeric G proteins.

APP source	G-protein	Citation
APP_695_ wt; peptide 20 (H_657_-L_676_	Gαo^∗^; ***not*** Gαs, Gαi_1, 2, 3,_	Nishimoto et al., 1993
Peptide 20 (H_657_-L_676_)	Gαo/i; ***not*** Gαs	Colombo et al., 1994; Lang et al., 1995
APP_695_ wt	Gαo^∗^; ***not*** Gαi_2_	Okamoto et al., 1995
APP_695_ wt	Gαo^∗^	Okamoto et al., 1996
APP_695_ wt, V_642_I, V_642_F, V_642_G	Gαo^∗^#; ***not*** Gαs, Gαi_2,_ Gαz	Ikezu et al., 1996
APP_695_ V_642_I, V_642_F, V_642_G	Gαo#; ***not*** Gαi_2_	Yamatsuji et al., 1996a
APP_695_ V_642_I, V_642_F, V_642_G	Gαo; ***not*** Gαi_2_	Yamatsuji et al., 1996b
APP_695_ V_642_I	Gαo#; ***not*** Gαt	Giambarella et al., 1997
APP_695_ wt	Gαo^∗^#; ***not*** Gαi_2_ or Gαs^#^	Brouillet et al., 1999
APP_695_ V_642_I	Gαo/i^†^	Hashimoto et al., 2000
APP_695_ wt, V_642_I	Gαo/i^†^	Sudo et al., 2000
APP_695_ wt, V_642_I	Gαo/i^†^	Niikura et al., 2000
APP_695_ wt	Gαo/i^†^	Mbebi et al., 2002
APP_695_ wt	Gαo; ***not*** Gαi_1_	Hashimoto et al., 2003a
EGFR-APP_icd_ chimera	Gαo/i^†^	Hashimoto et al., 2003b
APP_695_ V_642_I, APP_695_ KM_595-6_NL	Gαo	McPhie et al., 2003
APP_695_ wt, V_642_I	Gαo/i^†^	Niikura et al., 2004
APP_695_ wt	Gαo/i	Xu et al., 2009
APP_695_ wt	Gαo; ***not*** Gαi_2,_ Gαi_3,_	Sola Vigo et al., 2009
APP_695_ wt	Gαo^†^	Shaked et al., 2009
APPL (Manduca, Drosophila)	Gαo^∗^; ***not*** Gαi, Gαs	Ramaker et al., 2013
APP_695_ wt	Gαo^∗^; ***not*** Gαs	Ramaker et al., 2013
APP_695_ wt	Gαo^∗^	Fogel et al., 2014
APP_695_ wt	Gαo/i^†^	Milosch et al., 2014
APPL (Manduca)	Gαo	Ramaker et al., 2016a
Membrane-tethered AICD	Gαs^∗∗^	Deyts et al., 2012


*Summary of published evidence that APP interacts with Gαo (but usually not other G proteins, including Gαs, Gαz, and Gαi isofroms). The table includes studies on both wild type APP_695_, isolated peptide 20 constructs (containing the G protein-binding domain of APP_695_), and FAD-associated mutant forms of APP with altered residues at V_642_ (indicated in the left-hand column). Studies that showed direct binding between Gαo and APP/APPL are indicated with an asterisk (^∗^). Studies that used PTX to indicate the involvement of Gαo/i family proteins are indicated with a cross (\dagger). Studies that used CTX to indicate the absence of Gαs-dependent signaling is indicated with a hash mark (#). Study that showed direct binding between Gαs and constructs containing the G protein-binding domain is indicated with a double asterisk (^∗∗^). Citations for each set of results are shown in the right-hand column. ^∗^Studies that showed direct binding between Gαo and APP/APPL (or the G protein-binding domain). ^†^Studies that demonstrated sensitivity to PTX, indicating the involvement of Gαo/i. #Studies that tested sensitivity to CTX, indicating the absence of Gαs-dependent signaling. ^∗∗^Study that showed direct binding between Gαs and peptides containing the G protein-binding domain.*

In related experiments, Ikezu et al. (1996) co-expressed APP with chimeric Gα subunits to demonstrate that the last five amino acids of Gαo are necessary for its interactions with APP, whereas chimeras containing the cytoplasmic domains of other Gα subunits were ineffective (**Table 1**). This result is consistent with extensive evidence that C-terminal residues within Gα subunits control the specificity of their interactions with conventional GPCRs (Hamm et al., 1988; Herrmann et al., 2004). In collaboration with other groups, they also showed that soluble peptide 20 could regulate Go-dependent exocytosis but had no effect on Gs-dependent membrane fusion events, further validating the model that APP specifically interacts with the C-terminal region of Gαo (Colombo et al., 1994; Lang et al., 1995). These results provide strong evidence that the juxtamembrane G protein-binding domain in APP promotes functional interactions with Gαo (but not other G proteins), suggesting that APP might indeed function as an atypical Go-coupled receptor.

Subsequent studies explored whether stimulating APP with an antibody against its extracellular domain (22C11; to mimic ligand binding) could induce Gαo activity. In liposomes containing reconstituted APP_695_ and bovine Go, treatment with 22C11 induced the activation of Go (but not Gi_2_) in the absence of other proteins (Okamoto et al., 1995, 1996). Although the 22C11 antibody can also detect APLP2 (Slunt et al., 1994), other antibodies targeting different epitopes in APP (but not APLP1 or APLP2) were also found to induce Go-associated responses, including α-1680 and Alz90 (Sudo et al., 2000). In this regard, several groups also tested whether the effects of APP on Gαo signaling might be recapitulated by APLP1 or APLP2. Although one study showed that antibody activation of either APP or APLP2 could induce similar cytotoxic responses to 22C11 (Mbebi et al., 2002), other investigators used APP knockout lines to show that only re-expression of APP rescued Gαo-dependent responses, whereas expression of APLP1 and APLP2 did not (Sola Vigo et al., 2009; Milosch et al., 2014). Thus, these studies provided intriguing evidence that only APP can function as an unconventional Go-coupled receptor, albeit under rather artificial conditions.

## Aberrant APP-Go Signaling Can Provoke Neurodegeneration

How might the misregulation of normal APP-Go signaling contribute to the pathology of AD? To address this issue, Yamatsuji et al. (1996a,b) used COS cells expressing Go to compare the responses elicited by wild type APP_695_ versus APP containing missense mutations that are known to cause early onset FAD. In contrast to wild type APP_695_, expression of these “FAD-APP” mutant isoforms (including V_642_I, V_642_F, V_642_G) induced a dramatic increase in DNA fragmentation and apoptosis. This effect was blocked by PTX treatment (indicating Gαo/i proteins) or by expressing a dominant-interfering form of Gαo (**Table 1**), but was not affected by CTX (an activator of Gαs) and was absent in COS cells lacking Go. Notably, treatment with either synthetic Aβ_40_ or Aβ_42_ did *not* induce apoptotic responses in this assay, nor did conditioned medium harvested from cell cultures expressing the V_642_ mutant isoforms (which produce abundant Aβ_42_). In combination, these studies supported the model that mutated forms of APP linked with FAD can indeed function as constitutively active Go-coupled receptors. Moreover, they suggested that the pathophysiological effects of FAD-APP mutations might be caused by aberrant hyperactivation of Go-dependent signaling, rather than simply promoting the accumulation of neurotoxic Aβ. An appealing corollary to this model is that the downstream pathways regulated by Go could provide novel biomarkers or therapeutic targets for treating AD.

Unfortunately, attempts to identify these downstream pathways produced paradoxical results. For example, using COS cells co-expressing chimeric Gα subunits with different variants of APP, Ikezu et al. (1996) found that FAD-APP isoforms inhibited cAMP response element (CRE)-mediated transcription in a Gαo-specific manner. Curiously, this effect was independent of adenylyl cyclase (AC) activity, while inhibitors of Gβγ signaling (rather than Gαo) blocked apoptotic responses in this assay (Giambarella et al., 1997). From these studies, the authors concluded that APP signaling normally regulates both Gαo- and Gβγ-dependent pathways, whereby Gαo regulates CRE-dependent transcriptional responses, while Gβγ regulates other effectors (as yet undefined) that can induce apoptosis when chronically activated. More perplexing were the results from another group, who found that 22C11 treatment in brain membrane fractions actually *inhibited* Gαo-dependent responses (Brouillet et al., 1999), leading to the proposal that unknown proteins expressed by neurons but not glial-derived cells (or in reconstituted membranes) might regulate Gαo activation by APP (Brouillet et al., 1999; Sudo et al., 2000). How the misregulation of Gαo- versus Gβγ-dependent pathways might contribute to AD remained an open question.

## Neurotoxic Mechanisms of Misregulated APP- Gαo Signaling: Conflicting Models

Subsequent investigations have generated an unexpectedly complicated (and often contradictory) view of how the APP-Go pathway might function in the diseased nervous system. Using a variety of transfected cell lines, Nishimoto et al. (1993) first confirmed that the induction of APP-Gαo signaling (by antibody crosslinking or induced dimerization) required transmembrane APP (Sudo et al., 2000; Hashimoto et al., 2003a), and that hyperactivation of this pathway could induce apoptotic responses in cultured mouse neurons (see also Rohn et al., 2000). Both groups described classic features of neuronal apoptosis in their assays, including neurite degeneration, nuclear condensation, internucleosomal DNA cleavage, and activation of pro-apoptotic caspases (including caspase 3, 7, and 9). Treatment with inhibitors of glutathione metabolism or NADPH oxidase (as well as incubation with antioxidants) effectively blocked the cell death response, suggesting that hyperstimulation of the APP-Gαo pathway induces a chronic elevation of reactive oxygen species (ROS), resulting in the induction of caspase-dependent apoptosis. Moreover, expressing FAD-APP isoforms induced the same cytotoxic responses caused by hyperstimulating wild type APP, including activation of ASK1 (Apoptosis Signal-Regulating kinase) and its downstream effector JNK that resulted in chronic upregulation of NADPH oxidase, elevated ROS levels, and activation of pro-apoptotic caspases (Hashimoto et al., 2003b; Niikura et al., 2004). A similar response could be induced by expressing a chimeric protein containing the dimerization domain of the EGF receptor fused with the APP cytoplasmic domain, providing a plausible explanation for how the hyperstimulation of normal APP-Go signaling with crosslinking antibodies could provoke neuronal death in an Aβ-independent manner. By comparison, the neurotoxic effects of FAD-associated mutations within a different region of APP (K_595_/M_596_) were found to be independent of Go, suggesting that different disease-associated mutations in APP might perturb a variety of signaling pathways that affect neuronal viability (Hashimoto et al., 2000). Collectively, these results bolstered the argument that the aberrant APP-Go signaling might contribute to both late-onset AD and some forms of FAD.

However, it should be noted that enforced dimerization of APP (with crosslinked antibodies or chimeric fusion proteins) involves rather artificial methods that may not recapitulate authentic physiological or pathophysiological interactions. Moreover, it is difficult to reconcile these results with more recent evidence that ∼65% of membrane-bound APP in healthy cells is normally present in a dimeric configuration (Gralle et al., 2009). Nevertheless, these cytotoxic effects could be recapitulated by overexpressing an FAD-APP isoform (V_642_I-APP) in both neuroblastoma cells and primary neurons (Niikura et al., 2000, 2004), independent of Aβ-associated toxicity (Sudo et al., 2001). Alternatively, other groups have suggested that forced dimerization of APP might provoke Go-dependent apoptotic responses via a variety of other pathways, including PAK3-dependent re-entry into the cell cycle (McPhie et al., 2003), misregulation of Src-dependent actin dynamics and focal adhesion turnover (Xu et al., 2009), and calpain/calcineurin-dependent proteolysis of CaMKIV, resulting in the misregulation of CREB (Mbebi et al., 2002). Also problematic is the mechanism by which the APP-Go pathway might actually stimulate JNK: although both the α and βγ subunits of a number of heterotrimeric G proteins (including Go) can modulate JNK activity in different contexts, these responses typically require a cascade of other kinases and adapter proteins that have not been implicated in APP-Go signaling (Goldsmith and Dhanasekaran, 2007; Bromberg et al., 2008; Yu et al., 2016). Lastly, all of these studies focused on pathological outcomes that could be induced by aberrant APP-Gαo signaling, but the authentic functions of this pathway in the healthy nervous system remained largely unexplored. As described below, recent studies from the Kögel laboratory have now indicated that APP-Gαo signaling may actually *antagonize* the JNK pathway under physiological conditions, whereby the induction of APP signaling counteracts cellular stress responses via the PI3K cascade, providing a mechanism that promotes neuronal survival (Kögel et al., 2012; Milosch et al., 2014).

## Is APP-Gαo Signaling Altered in Human Patients with AD?

Whether the misregulation of APP-Go signaling actually plays a role in provoking AD remains unknown. However, a variety of studies have offered intriguing hints that support this hypothesis. Initial reports using human brain samples revealed that the expression patterns of many heterotrimeric G proteins are altered in late sporadic AD, particularly within the most vulnerable brain regions (including cortex and hippocampus). These changes also correlate with a general reduction in G protein-dependent GTP hydrolysis at stages that precede the onset of clinical disease (O’Neill et al., 1994; Cowburn et al., 2001; Garcia-Jimenez et al., 2002). Similarly, using reconstituted membrane preparations from human brain samples, Mahlapuu et al. (2003) found that the induction of G protein activity by APP-derived peptides was significantly reduced in post-mortem elderly AD patients compared to age-matched controls. Recapitulating the original studies by Nishimoto et al. (1993), they also found that membrane-tethered constructs of the Go domain (peptide 20 plus the transmembrane T_639_-L_649_ sequence) induced more robust [^35^S]GTPγS binding than soluble peptide 20 (Mahlapuu et al., 2003). Curiously, adding the transmembrane peptide alone (T_639_-L_649_) also affected [^35^S]GTPγS binding, while equivalent peptides containing V_642_ APP-FAD mutations were even more effective (Karelson et al., 2005), although how these hydrophobic constructs might interact with G proteins when applied to isolated membranes is unclear. Nevertheless, these results provided indirect evidence that disease-associated changes in the GPCR-like function of APP might contribute to both FAD and late-onset AD (as noted by the authors).

Perhaps because it is the most abundant G protein in the brain (Strittmatter et al., 1990; Jiang and Bajpayee, 2009), the overall levels of Gαo do not appear to be altered in either FAD or late-onset sporadic AD (O’Neill et al., 1994; Shaked et al., 2009), but several studies suggest that Gαo-specific responses are progressively disrupted in both familial and late sporadic forms of the disease. For example, using membrane preparations from human brain samples, Reis et al. (2007) found that the effects of FAD-APP-derived peptides on G protein activity were blocked by PTX, while another report showed that Aβ peptides could activate Gαo in lipid vesicles (Rymer and Good, 2001), although it is unclear whether the topology of these assays recapitulates authentic Gαo-Aβ interactions. More compelling are two studies showing that APP-Go signaling might be directly altered by neurotoxic Aβ in neurons. Based on previous evidence that APP can bind neurotoxic Aβ fibrils (Lorenzo et al., 2000; Van Nostrand et al., 2002), Lorenzo et al. (2000) also showed that APP overexpression rendered hippocampal neurons more vulnerable to Aβ-induced degeneration, an effect that was abrogated by deletion of the Go-binding domain in APP or treatment with PTX (Sola Vigo et al., 2009). Notably, expressing a PTX-insensitive form of Gαo restored the toxic effects of Aβ treatment, but only in the presence of an intact Go-binding domain. Subsequent work by Masliah and colleagues demonstrated that treatment with Aβ reduced APP-Gαo interactions (corresponding to Go activation) and induced cell death in transfected neuroblastoma lines, and again this effect was PTX-dependent (Shaked et al., 2009). Aβ treatment also provoked a significant increase in calcium (Ca^2+^) influx in a Go-dependent manner, consistent with earlier studies suggesting that hyperactivation of APP signaling could provoke Ca^2+^ overload and cell death. Most notably, they showed that APP-Gαo interactions declined in patients suffering from progressive stages of AD, corresponding to an overall increase in G protein activation (though not specifically Gαo).

In the course of their cell culture assays, the authors found that mutating a particular residue within the cytoplasmic domain of APP (D_664_A) blocked the ability of Aβ to affect APP-Gαo interactions (Shaked et al., 2009). Noting that this residue is required for caspase-dependent cleavage of APP to generate a cytotoxic C31 fragment (Lu et al., 2003), they proposed a mechanism by which Aβ binding induces caspase-dependent cleavage of APP, resulting in the release of a C31-Go complex that could stimulate Gαo in some undefined fashion. However, other investigators have noted that the D_664_A mutation (located within the Go domain) is equally likely to disrupt interactions between APP and other cytoplasmic proteins (Galvan et al., 2007), the most obvious candidate being Gαo. Thus, mutations at this site might perturb key structural features that permit APP to function as a Go-coupled receptor, although the steric rearrangements that lead to the activation of Gαo remain unexplored. Paradoxically, Shaked et al. (2009) also reported that deletion of the C-terminal YENPTY domain mitigated the effects of Aβ on Gαo activation, contradicting several previous studies demonstrating that this motif is *not* required for direct interactions between APP and Gαo (Nishimoto et al., 1993; Kawasumi et al., 2004; King and Scott Turner, 2004; Sola Vigo et al., 2009). Nevertheless, these results offered the most compelling evidence that APP-Go signaling is altered over the course of AD, consistent with the model that elevated Aβ might induce the aberrant activation of Gαo-dependent pathways that provoke neuropathological responses.

Recently, Fogel et al. (2014) used fluorescence resonance energy transfer (FRET)-based protocols to demonstrate a close association between APP and Gαo that was modulated by APP activation. They also showed that Aβ_40_ induced structural rearrangements in the presynaptic APP/Go complex by promoting APP dimerization, which in turn resulted in G protein-dependent Ca^2+^ influx and glutamate release (Fogel et al., 2014). Both aspects of this response were found to critically involve the E1 extracellular domain of APP, suggesting that Aβ_40_ can mimic the effects of endogenous ligands. Based on these findings, the authors proposed that excessive APP activation by amyloid peptides might contribute to hippocampal hyperactivity under pathological conditions, supporting the hypothesis that normal APP-Gαo interactions are altered in AD. An added dimension to this model is that Gαo may also functionally interact with presenilins, essential components of the γ-secretase complex that are involved in generating Aβ peptides and AICD fragments and are also mutated in some forms of FAD (Walter et al., 2001; Jayne et al., 2016). For example, Smine et al. (1998) showed that presenilin-1 (PS-1) could be co-immunoprecipitated with Gαo (but not Gαi_2_) when overexpressed in COS-7 cells, and that a C-terminal fragment (CTF) of PS-1 could activate Gαo (but not Gαi_2_) in a PTX-sensitive manner. Likewise, overexpressing FAD mutant forms of Presenilin-2 (PS-2) in neuroblastoma cells induced apoptotic responses that were inhibited by PTX and restored by expressing a PTX-resistant variant of Gαo but not Gαi (Wolozin et al., 1996; Abe et al., 2004). Whether presenilins actually modulate Gαo-dependent pathways in neurons and how this might affect APP-Gαo interactions remains to be explored. Nevertheless, it is possible that multiple factors associated with AD might contribute to the pathological misregulation of APP-Gαo signaling (including FAD-linked mutations in both APP and the presenilins), as well as the accumulation of neurotoxic amyloid peptides that can hyperactivate this pathway.

## Structure, Specificity, and Evolutionary Conservation of the Go-BINDING Domain in APP Family Proteins

As noted earlier, Nishimoto et al. (1993) first identified the G protein-binding domain in APP, based on their previous discoveries that several type-1 transmembrane proteins directly bind Gα subunits via short peptide sequences containing BBXB or BBXXB motifs, where B is a basic amino acid residue and X is any non-basic residue (Okamoto et al., 1990, 1991; Okamoto and Nishimoto, 1992). From this analysis, they identified “peptide 20” in APP_695_ (H_657_-L_676_), which contains two N-terminal basic residues (HH) and terminates in a BBXXB motif (**Figure 1A**; magenta region). In a meticulous series of experiments using reconstituted liposomes and isolated membrane fractions, they then showed that this “peptide 20” domain (subsequently designated the Go activator domain) was both necessary and sufficient for directly binding and activating Gαo, but *not* Gαs, Gαi_1_, Gαi_2_, or Gαi_3_ (**Table 1**). Removing either the N-terminal histidines (**Figure 1A**, asterisks) or the C-terminal BBXXB motif from peptide 20 (RHLSK) greatly attenuated its ability to simulate Gαo in GTPase activation assays, although membrane-tethered versions of the Go domain were considerably more potent than soluble forms. Thirdly, they demonstrated that interactions between full-length APP and Gαo required this domain: a deletion that removed both the Go domain and the C-terminal YENPTY motif precluded APP-Gαo interactions (His_657_-N_695_; **Figure 1B_1_**), whereas a deletion encompassing only the YENPTY did not (**Figure 1B_2_**). These results provide strong evidence that the juxtamembrane G protein-binding domain in APP promotes functional interactions with Gαo but not other G proteins (Nishimoto et al., 1993).

Using similar methods, Nishimoto et al. (1993) subsequently showed that full-length APP binds and stimulates Gαo (but not Gαi_2_) following antibody activation in reconstituted vesicles (Okamoto et al., 1995; Ikezu et al., 1996), while the apoptotic effects of FAD-APP isoforms (mutated at V_642_) were both PTX-sensitive and required the Go domain: FAD-APP constructs lacking only the Go domain (**Figure 1B_3_**) failed to induce Gαo-dependent cytotoxic responses, whereas deletions encompassing the YENPTY domain (**Figure 1B_2_**) had no effect (Okamoto et al., 1996; Yamatsuji et al., 1996a; Hashimoto et al., 2000; Niikura et al., 2000; Sudo et al., 2001). This apoptotic response could also be blocked with dominant-interfering forms of Gαo (GαoG204A) but not Gαi_2_ (**Table 1**; Yamatsuji et al., 1996b). Using Myc-tagged constructs for *in vitro* pull-down assays, Brouillet et al. (1999) subsequently confirmed that the cytoplasmic domain of APP could bind Gαo but not Gα_i2_, and that this interaction was reduced when the N-terminal H_657_H_658_ doublet was replaced with hydrophobic residues. Sudo et al. (2001) and Hashimoto et al. (2003a) then showed that that apoptotic effects of APP stimulation were prevented by deleting the Go interaction domain (**Figure 1B_3_**) but not the YENPTY domain (**Figure 1B_2_**), and that they were mediated specifically by Gαo but not Gαi. Similarly, based on evidence that Aβ might induce neurotoxic responses via the APP-Gαo pathway, Lorenzo and colleagues showed that this effect also required the Go domain (Sola Vigo et al., 2009): deleting the entire cytoplasmic domain (**Figure 1B_4_**) precluded the activation of Gαo-dependent responses to Aβ, as did complementary deletions targeting different portions of the Go domain (**Figure 1B_5_,_6_**), whereas a deletion encompassing the YENPTY motif did not (**Figure 1B_7_**). In a more physiological context, the Kögel group recently demonstrated the importance of the Go domain in mediating APP-dependent neuroprotective responses to sAPPα: a deletion that removed the conserved PEERH motif within this domain (**Figure 1B_9_**) prevented APP-dependent signaling that was also blocked by PTX (implicating Gαo/i proteins), whereas two different deletions targeting the YENPTY motif (**Figure 1B_10_,_11_**) had no effect (as summarized below).

In contrast to the foregoing studies, Shaked et al. (2009) reported that Gαo could still be co-immunoprecipitated with APP lacking the C31 cytoplasmic region (including both the Go-binding domain and the YENPTY motif; **Figure 1B_12_**), but that deleting this region prevented APP-dependent activation of Gαo pathways in cell culture. They also found that over-expressed C99 fragments could be co-immunoprecipitated with Gαo (the only report of this interaction). Curiously, deletion of only the YENPTY motif (**Figure 1B_13_**) also blocked Gαo-dependent responses in this assay, in contrast to many other studies demonstrating that this domain is not required for APP-Gαo interactions. Based on these observations, the authors postulated that the transduction of APP-Gαo signaling might involve the YENPTY motif as well as the Go domain (either directly or indirectly), possibly in response to Aβ-induced cleavage of APP (Shaked et al., 2009). Whether this response also involves internalization responses mediated by the YENPTY motif remains to be explored (cf. Lai et al., 1995; Deyts et al., 2016b).

Other members of the APP family also contain Go-like domains, albeit with some sequence variations (**Figure 1C**). Both APLP1 and APLP2 contain only one N-terminal histidine that aligns with the HH doublet in APP_695_ (highlighted in yellow), and only APLP2 also possesses an intact C-terminal BBXXB motif (boxed region). As summarized above, only APP_695_ has been shown to activate Gαo, although a rigorous analysis of potential interactions between APLP1/2 and Gαo has not been conducted *in vivo*. Likewise, the Go domains in both nematode APL-1 and insect APPL contain only a single N-terminal histidine and lack complete BBXXB motifs. Nevertheless, studies in several insect models have shown that APPL does functionally interact with Gαo both *in vitro* and *in vivo*, whereby deleting different portions of the Go domain in APPL (**Figure 1D**_1_,_2_) disrupted Gαo-associated responses in the developing nervous system (Torroja et al., 1999b; Ramaker et al., 2013; and described below). How these structural variations within the Go domain might affect the dynamics of Gαo activation/inactivation under physiological conditions remains to be explored.

## Physiological Role of APP-Gαo Interactions in Stress Signaling and Neuroprotection

Based on early work suggesting that APP might regulate both cell adhesion and excitoprotective responses (Mattson et al., 1993; Schubert and Behl, 1993), a variety of *in vitro* and *in vivo* assays demonstrated that both full-length APP and its sAPPα ectodomain fragments (produced by α-secretase cleavage) could have potent neuroprotective activity under different conditions (reviewed in Kögel et al., 2012; Nhan et al., 2015). For example, deletion of the sole APP ortholog in nematode (APL-1) caused larval lethality that could be rescued by expressing extracellular domain fragments equivalent to sAPPα (Hornsten et al., 2007; Ewald et al., 2016), while overexpressing sAPPα rescued some behavioral deficits in mice lacking members of the APP family (Ring et al., 2007; Weyer et al., 2011). From these and other experiments emerged a complex scenario whereby both APP and sAPPα might independently confer beneficial responses under physiological conditions. However, elevated sAPPα levels can also have unwanted effects on cell proliferation and tumorigenesis, potentially due to interactions with receptors whose roles in neuroprotection is unclear (Adlerz et al., 2007; Zhou et al., 2011). More recently, Kögel and colleagues have provided new evidence that transmembrane APP and sAPPα interact as a ligand/receptor pair in neurons to modulate stress signaling, via activation of the pro-survival PI3K/Akt pathway (Milosch et al., 2014). Using a variety of experimental strategies, they demonstrated that both APP and sAPPα antagonize the activation of the JNK-dependent stress signaling pathway, which (as noted earlier) is a key upstream modulator of mitochondria-dependent apoptosis (Kögel et al., 2005; Copanaki et al., 2010; Eckert et al., 2011). Conversely, several groups have now shown that the protective function of APP requires activation of the PI3K/Akt pathway (Cheng et al., 2002; Copanaki et al., 2010; Eckert et al., 2011; Jimenez et al., 2011). Since Akt negatively regulates several JNK-activating kinases, including ASK1 and mixed lineage kinase 3 (MLK3), these findings suggest that APP modulates a dynamic interplay between stress and survival pathways (Kögel et al., 2012).

To define the role of full-length APP in this response, Milosch et al. (2014) showed that the protective effects of both sAPPα and a recombinant fragment containing only the E1 domain of APP were completely abrogated in neurons from APP knockout animals or in APP-depleted SH-SY5Y cells. These results clearly demonstrated that expression of membrane-bound holo-APP was required for sAPPα-dependent Akt activation and neuroprotection in these assays, supported by other evidence that sAPPα can regulate the dimerization of transmembrane APP in cell culture (Gralle et al., 2009; Kaden et al., 2012). Likewise, studies in *Drosophila* have shown that sAPPL ectodomain fragments (equivalent to sAPPα) bind full-length APPL, and that the neuroprotective effects of sAPPL require the presence of the holoprotein (Wentzell et al., 2012). More recently, a behavioral analysis demonstrated that full-length APPL and secreted sAPPLα act together to promote memory formation in adult *Drosophila* (Bourdet et al., 2015), consistent with the model that APP-sAPPα interactions may serve a variety of physiological functions in the nervous system.

Although the foregoing experiments demonstrated that the C-terminal domain of APP was required for the neuroprotective effects of the holoprotein, the last 15 amino acids were dispensable (as summarized in **Figure 1B_8-11_**): sAPPα-dependent activation of Akt was unaffected in neurons from APP-ΔCT15 mice, which express a mutant form of APP lacking the cytoplasmic YENPTY motif (Milosch et al., 2014). As noted in other reviews, this domain mediates interactions with a plethora of cytoplasmic proteins but not Gαo (Nishimoto et al., 1993; King and Scott Turner, 2004; Sola Vigo et al., 2009). To further map the specific regions in APP that are required for this activity, APP-KO cells were transfected with an APP construct lacking the PEER motif within its Go-binding domain (ΔPEERH). In contrast to the YENPTY mutant, the ΔPEERH mutant did not rescue sAPPα-induced Akt activation following trophic factor deprivation. In addition, treatment with PTX completely abolished the ability of sAPPα to promote Akt activation and cell survival, further implicating a role for Go in this response. Lastly, activation of the PI3K/Akt pathway by sAPPα induced the phosphorylation of glycogen synthase kinase 3β (GSK3β), which is a well-known mechanism for inhibiting GSK3β-induced apoptotic responses (Watcharasit et al., 2003; Hanumanthappa et al., 2014). Whereas PI3K/Akt signaling was originally linked with receptor tyrosine kinase activation, numerous studies have shown that heterotrimeric G proteins also play a critical role in regulating PI3K activity under both physiological and pathological conditions (Murga et al., 1998; Murga et al., 2000; New and Wong, 2007; Yanamadala et al., 2009). Since PTX selectively inhibits members of the Gαo/i family, while APP only interacts with Gαo and potentially Gαs (as noted below), these results argue that APP/sAPPα interactions induce the PI3K/Akt pathway specifically via Gαo.

**FIGURE 2:**
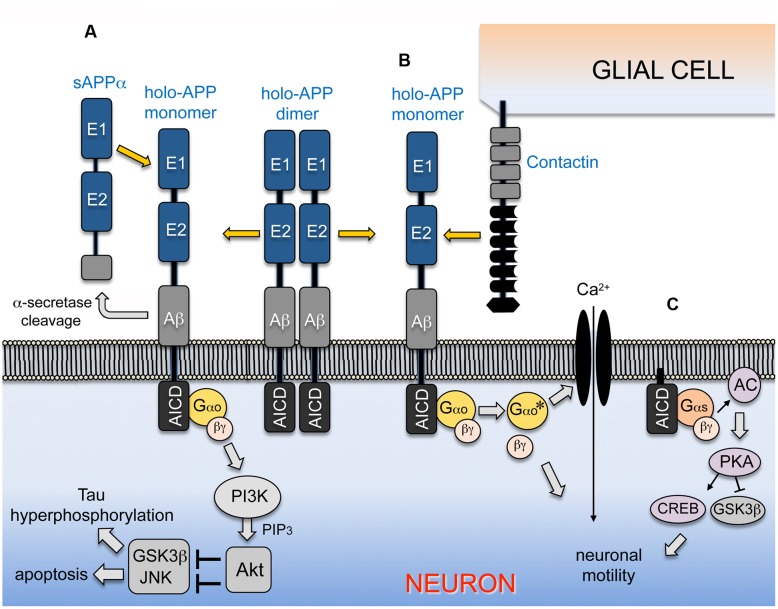
**APP-Go signaling can regulate alternative downstream pathways in a context-dependent manner.** **(A)** APP is inserted into the plasma membrane of neurons as a type-1 transmembrane protein that directly interacts with the heterotrimeric G protein Go; the major fraction of the holoprotein spontaneously forms homodimers under unstimulated conditions. Interactions with sAPPα ectodomain fragments (generated by α-secretase processing) promotes the dissociation of homodimeric APP and activates Gαo, stimulating the exchange of bound GDP for GTP on the Gαo subunit and its dissociation from the Gβγ dimer (similar to signaling by conventional GPCRs; Fogel et al., 2014). Both activated Gαo and Gβγ may stimulate PI3K, which then phosphorylates and activates Akt. In turn, Akt phosphorylates and inhibits downstream targets linked with apoptotic responses and Tau hyperphosphorylation, including GSK3β and components of the stress kinase pathway that regulate JNK (Kögel et al., 2003; Copanaki et al., 2010). In this manner, stimulation of the APP-Gαo pathway by sAPPα promotes neuroprotective responses by modulating neuronal stress signaling, providing a mechanism for integrating the stress and survival responses regulated by APP and its cleaved sAPPα ectodomain fragments (Kögel et al., 2012; Milosch et al., 2014). **(B)** In the developing nervous system of *Manduca*, migratory neurons co-express insect APP (APPL) and Gαo in their leading processes (Swanson et al., 2005; Ramaker et al., 2013), while their ensheathing glial cells express a single Contactin ortholog (MsContactin). Embryo culture assays have shown that glial Contactin stimulates APP-Go signaling in the migratory neurons, whereby Gαo-dependent induction of Ca^2+^ currents (and possibly other effectors regulated by via Gβγ) induces local retraction responses that prevent ectopic migration and outgrowth (Horgan and Copenhaver, 1998; Ramaker et al., 2016b). **(C)** Membrane-tethered AICDs and APP-CTFs can also interact with Gαs to stimulate neuronal motility and outgrowth, via a pathway that involves the activation of adenylate cyclase/cAMP/PKA/CREB signaling, accompanied by the phosphorylation/inactivation of GSK3β (Deyts et al., 2012, 2016a). Stimulation of APP signaling by different combinations of ligands and co-receptors might preferentially activate Gαo- or Gαs- associated responses in a context-dependent manner, whereby APP-G protein signaling can either promote or inhibit neuronal motility at specific stages and locations in the nervous system.

Based on these findings, we propose that transmembrane APP mediates sAPPα-induced neuroprotection via Gαo-coupled activation of the PI3K/Akt pro-survival pathway (**Figure 2A**). In turn, activation of Akt phosphorylates and inhibits GSK3β, as well as other pro-apoptotic targets (Datta et al., 1997; Endo et al., 2006; Jover-Mengual et al., 2010). We also propose that this response requires direct interactions between sAPPα and holo-APP as a ligand-receptor pair. These results offer a resolution to paradoxical findings from previous investigations, demonstrating that holo-APP and sAPPα are equally important in mediating neuroprotective responses. Conversely, factors that interfere with this function would render neurons more susceptible to cellular stress during brain aging and AD. The model that APP-Gαo signaling serves a neuroprotective function under physiological conditions contrasts with the cytotoxic response elicited by hyperactivating this pathway in AD models (as summarized above). Of note is that treatment with Aβ might also interfere with the neuroprotective effects of sAPPα, resulting in the disinhibition of GSK3β and consequent upregulation of apoptotic pathways (Jimenez et al., 2011). Since GSK3β activity is increased in the AD brain (Crews and Masliah, 2010; Jimenez et al., 2011; Llorens-Martin et al., 2014), we hypothesize that the decline in sAPPα levels associated with both sporadic AD and FAD contributes to this phenomenon (Almkvist et al., 1997; Sennvik et al., 2000), thereby promoting tau hyperphosphorylation (Deng et al., 2015) and sensitizing neurons to stress and apoptosis. In summary, these studies provide new insight into the mechanisms by which APP-Go signaling regulates neuronal stress responses under physiological conditions, and how the loss of this function might render neurons more susceptible to cellular stress during normal brain aging and AD.

## APP-Gαo Signaling in the Control of Neuronal Motility: Views From A Non-Mammalian System

Although APP was originally identified in humans, it is actually a member of an evolutionarily ancient family of proteins that may serve similar roles in the developing nervous systems of many organisms (Coulson et al., 2000; Ewald and Li, 2012; Lazarov and Demars, 2012; Shariati and De Strooper, 2013). Studies using a variety of insect models have shown that APPL shares both structural and functional conservation with human APP_695_, including homologous extracellular and intracellular motifs that regulate interactions with other proteins (Cassar and Kretzschmar, 2016). In particular, several groups have demonstrated a role for APPL-Gαo signaling in neuronal development. Using genetic methods, Torroja et al. (1996, 1999a) first showed that APPL plays an important role in regulating neuronal growth and maturation, and that this activity requires the conserved Go-binding domain shared by APP_695_ and APPL. Replacing endogenous APPL with a mutant form lacking this domain (**Figure 1D_1_**) disrupted the normal maturation of synaptic boutons at the neuromuscular junction, potentially caused by the loss of ligand-dependent APPL-Go signaling (Torroja et al., 1999b). Subsequent investigations into this response suggested a role for the homophilic cell adhesion receptor Fasciclin II (Fas II; the insect ortholog of NCAM), whereby trans-synaptic interactions mediated by Fas II could promote APPL signaling, in part via the activation of Gαo. Whether Fas II acts as a ligand as well as a co-receptor for APPL remains to be explored, as does the role of downstream Gαo effectors in regulating synaptic maturation. Nevertheless, this work offered compelling evidence that the APP-Go pathway is conserved in both invertebrate and vertebrate nervous systems.

Using *Manduca sexta* (tobacco hornworm) as a complementary model, the Copenhaver laboratory has also explored the role of APPL-Gαo signaling in the developmental control of neuronal motility. Unlike *Drosophila*, the formation of the embryonic nervous system in *Manduca* involves an extended period of neuronal migration (Copenhaver and Taghert, 1989; Copenhaver, 2007), analogous to the more complex waves of migration that typify mammalian brain development (Ayala et al., 2007; Tabata and Nagata, 2016). Notably, APPL colocalizes with Gαo in the leading processes and growing axons of migratory neurons in *Manduca* (Swanson et al., 2005), similar to the colocalization of APP and Gαo in cultured mammalian neurons (Ramaker et al., 2013). In addition, co-immunoprecipitation assays showed that endogenously expressed APPL and Gαo functionally interact in a manner that is regulated by Gαo activation (Ramaker et al., 2013). By co-expressing fusion constructs of APPL and Gαo containing complementary portions of Venus fluorescent protein in transfected COS7 cells, bimolecular fluorescence complementation (BiFC) assays were used to demonstrate that transmembrane APPL directly bound Gαo (but not Gαi or Gαs), while APP_695_ also directly bound Gαo, similar to conventional GPCRs (Marinissen and Gutkind, 2001; Oldham and Hamm, 2008). More importantly, expressing these constructs in transgenic *Drosophila* lines revealed that APPL bound Gαo in healthy neurons, providing the first demonstration of direct interactions between an APP family protein and Gαo *in vivo.* Notably, this interaction could be readily visualized within synaptic regions of the brain by BiFC, whereas deleting the Go domain in APPL (**Figure 1D**_2_) eliminated APPL-Gαo binding (Ramaker et al., 2013). In combination, these studies substantiate the model that APP family proteins can indeed function as unconventional GPCRs, specifically regulating Gαo-dependent responses.

By adapting an embryo culture assay that permits targeted manipulations of migratory neurons in *Manduca* (Horgan and Copenhaver, 1998), the Copenhaver laboratory subsequently showed that APPL-Gαo signaling plays an important role in regulating neuronal motile behaviors: inhibiting either APPL expression or Gαo activity induced a distinctive pattern of ectopic growth and migration, while hyperstimulating the APPL-Gαo pathway induced collapse-stall responses (Ramaker et al., 2013). These effects were analogous to the striking pattern of ectopic neuronal migration reported in the brains of mice deleted for all three APP family proteins (Herms et al., 2004), and recapitulated earlier studies in *Manduca* showing that activated Gαo inhibits migration via the induction of voltage-independent currents (Horgan et al., 1995; Horgan and Copenhaver, 1998). More recent studies have identified *Manduca* Contactin (MsContactin) as a candidate ligand for APPL (Ramaker et al., 2016b). Specifically, experiments in cultured embryos indicated that GPI-linked MsContactin (expressed by adjacent glial cells) activates APPL-Gαo signaling in the migratory neurons to induce local retraction responses (**Figure 2B**), thereby preventing ectopic outgrowth. This discovery was supported by reports that multiple Contactin family members in mammalian systems can interact with APP and its orthologs both in *cis* and *trans* (Ma et al., 2008; Osterfield et al., 2008; Tachi et al., 2010; Osterhout et al., 2015). In summary, our experiments provide new evidence that APP family proteins regulate key aspects of neuronal development during embryogenesis, in part via activation of Gαo-dependent pathways. Still to be determined are the downstream effectors that transduce the effects of APPL-Gαo signaling on neuronal behavior. Likewise, whether mammalian Contactins might regulate APP-Gαo signaling in migratory cortical neurons, and whether modulation of the PI3K-Akt pathway or GSK3β activity also contributes to this response within the developing nervous system remains to be explored (e.g., Morgan-Smith et al., 2014).

## APP May Also Regulate Neuronal Motility via Gαs-Dependent Pathways

Most studies support the model that transmembrane APP normally binds and activates Gαo in response to a variety of ligands (including sAPPα and MsContactin), suggesting that APP cleavage (by secretases or caspases) is likely to *terminate* APP-Gαo signaling rather than activating it. In support of this model, we recently showed that blocking α-secretase activity in the migratory neurons of cultured *Manduca* embryos significantly increased membrane-associated APPL levels, while inducing the same collapse/stall responses caused by hyperactivating APPL-Gαo signaling with Contactin fusion proteins (Ramaker et al., 2016a,b). Likewise, our analysis of endogenously expressed APP family proteins showed that Gαo could be readily co-immunoprecipitated with both full length APP_695_ (from mouse and human brain lysates) and APPL (from *Manduca* and *Drosophila* lysates), whereas we did not detect their CTF or AICD fragments in the immunoprecipitated complexes (Ramaker et al., 2013). These results are also consistent with past work focusing on the functional interactions between transmembrane APP_695_ and Gαo (e.g., Okamoto et al., 1995; Hashimoto et al., 2003a; Sola Vigo et al., 2009). However, as noted above, several reports have shown that Gαo can also interact with membrane-tethered peptide 20 domains (mimicking CTFs that contain the Go-binding domain), and one study showed that Gαo could be co-immunoprecipitated with C99 fragments (normally generated by β-secretase cleavage) when overexpressed in neuroblastoma cells (Shaked et al., 2009). Whether Gαo actually continues to interact with CTFs following α- or β-cleavage of the holoprotein in neurons, and whether these interactions might affect downstream pathways regulated by APP-Gαo signaling under physiological conditions, is still unknown.

In contrast, recent studies by Parent and colleagues have indicated that a different G protein (Gαs) may be *activated* by CTFs derived from the APP holoprotein (Deyts et al., 2012). Specifically, they found that overexpressing a membrane-tethered AICD construct (mAICD) or experimentally elevating intracellular APP-CTF levels dramatically increased neurite outgrowth in both neuroblastoma cells and transfected cortical neurons. This response required AC-dependent activation of protein kinase A (PKA) and corresponded to the phosphorylation of two PKA targets (CREB and GSK3β), both of which can regulate neuronal motility. To test the involvement of Gαs (a canonical activator of AC), they also showed that HA-tagged Gαs could be co-immunoprecipitated with mAICD from transfected cells, whereas dominant-negative Gαs (lacking its palmitoylation site) prevented mAICD-induced outgrowth. Focusing on the BBXXB motif in APP that was originally identified by Nishimoto et al. (1993) (**Figure 2C**, asterisks), Deyts et al. (2012) found that mutating this site prevented interactions between the mAICD construct and HA-Gαs. Curiously, they also demonstrated an interaction between Gαs and an equivalent construct derived from APLP1, which (like insect APPL) lacks a BBXXB motif (**Figure 1C**, boxed region), suggesting that this motif may not be strictly required for functional interactions between APP family proteins and Gα subunits within intact neurons.

More recently, the Parent group conducted a series of carefully controlled experiments in both cultured neurons and transgenic mice, demonstrating that elevating APP-CTF levels (by a variety of methods) induced exuberant neurite outgrowth, coincident with enhanced PKA and CREB phosphorylation (Deyts et al., 2016a). Consistent with their earlier work, they found that overexpressing β-CTF fragments of APP (C99) also stimulated outgrowth, whereas a C99 construct with a mutated BBXXB motif did not. Lastly, they showed that treatment with an AC inhibitor prevented increased outgrowth and phosphorylated CREB levels in their assays, again implicating Gαs-dependent signaling. Whether Gαs endogenously interacts with APP-CTFs in healthy neurons and whether this interaction is perturbed over the course of AD remains to be explored. Nevertheless, given available evidence that Gαo normally interacts with full-length APP but not its fragments in neurons (as summarized above), these results support the intriguing view that APP cleavage might induce a novel type of G protein switching (Tucek et al., 2002; Woehler and Ponimaskin, 2009), whereby the holoprotein signals as a transmembrane receptor specifically via Gαo, while its CTF fragments can selectively regulate Gαs-dependent pathways (**Figure 2C**). In the context of neuronal development, this model might also help explain how APP-dependent signaling can promote neuronal motility in some contexts while restricting it in others.

## Conclusion and Perspective: Ligand-Dependent Modulation of APP-Gαo Signaling

Despite considerable efforts to establish a role for aberrant APP-Gαo signaling in AD, proof for this model has been hindered by incomplete understanding of the mechanisms that normally regulate this pathway in the brain. Because past studies often relied on rather artificial assays and overexpression systems, it is still unclear whether hyperstimulating this pathway results in the misregulation of endogenous signaling responses or produces novel gain-of-function effects that normally do not occur in the brain. Our laboratories have now approached this issue using complementary strategies, with the goal of understanding how this evolutionarily conserved signaling pathway regulates neuronal functions in both the developing and mature nervous system. As summarized in **Figure 2A**, sAPPα ectodomain fragments are clearly able to activate the PI3K/Akt pathway and modulate neuronal stress signaling, a response that undoubtedly plays important roles in both the developing and adult brain (Kögel et al., 2012; Milosch et al., 2014). By comparison, Contactin-dependent activation of APP-Go signaling can regulate the motile behavior of developing neurons (**Figure 2B**), in part by modulating Ca^2+^ influx and downstream effectors that modulate cytoskeletal dynamics (Horgan and Copenhaver, 1998; Copenhaver and Ramaker, 2016). Evidence that CTF fragments might also regulate neuronal behavior via Gαs (**Figure 2C**) suggests that G protein switching could also contribute to the refinement of APP-dependent motile responses (Deyts et al., 2012, 2016a).

We postulate that our different experimental preparations have revealed an important aspect of APP-Go signaling: namely, that the integration of this pathway with alternative or complementary effectors can be strongly influenced by particular combinations of ligands and co-receptors for APP that are expressed in a context-dependent manner. As has been reviewed elsewhere, APP family proteins can interact with a wide variety of candidate binding partners (Hoe et al., 2009; Jacobsen and Iverfeldt, 2009; Rice et al., 2013; Deyts et al., 2016b), although most of these interactions have yet to be validated *in vivo*. For example, experiments using cultured neurons have shown that stimulation with sAPPα can *promote* APP-dependent outgrowth via interactions with members of the integrin and L1CAM families (Osterfield et al., 2008; Young-Pearse et al., 2008), a response that can be further modulated by extracellular proteins like Reelin, F-spondin, and Semaphorin 3A (Ho and Sudhof, 2004; Hoe et al., 2009; Magdesian et al., 2011). More recently, elegant work by Young-Pearse and colleagues showed that different members of the pancortin family can both promote and inhibit APP-dependent responses in migrating cortical neurons, possibly via a combination of direct and indirect interactions (Rice et al., 2012). Whether these interactions also regulate Go-dependent aspects of motility remains to be explored. Outside the nervous system, APP family proteins are strongly upregulated by keratinocytes during wound healing (Herzog et al., 2004), while treatment with sAPPα stimulates their motile behavior (Kirfel et al., 2002), although it is unclear if this response is transduced by APP or other receptors. From a developmental perspective, ample precedent for this model of APP-Go signaling can be found in the responses elicited by other neuronal guidance receptors that can both stimulate and inhibit outgrowth, depending on a variety of interacting factors (Nishiyama et al., 2003; Egea and Klein, 2007; Yoshida, 2012; Finci et al., 2014; Kaplan et al., 2014). Likewise, whether activation of APP-Gαo signaling induces neuroprotective or neurotoxic responses might be strongly affected by convergent input from physiological stimuli (particularly sAPPα) or pathological factors (including Aβ_42_ oligomers).

Lastly, it should be noted that APP expression is significantly altered in a variety of other diseases besides AD. In Down syndrome (DS), trisomy 21 results in a triplication of the gene encoding APP (as well as many other genes; Antonarakis et al., 2004), and most DS patients exhibit accelerated Aβ accumulation and develop AD-like neurological pathologies (Millan Sanchez et al., 2012; Castro et al., 2016). APP expression is also dramatically upregulated in the brain following traumatic brain injury (Plummer et al., 2016; Acosta et al., 2017) and in lesions associated with epilepsy and multiple sclerosis (Noebels, 2011; Matias-Guiu et al., 2016). Whether APP serves a neuroprotective function or promotes degenerative responses in these diseases is still unknown; hence, determining how APP-Gαo signaling is altered in AD should also be relevant to other conditions in which this pathway might be misregulated. Only by fully defining the normal mechanisms of APP-Go signaling in the brain will it be possible to resolve how the misregulation of this pathway may contribute to the pathological sequelae that give rise to AD.

## Author Contributions

PC and DK contributed equally to all aspects of this review, including development of the overall concept, writing and correcting the text, and creating the table and figures included in the review.

## Conflict of Interest Statement

The authors declare that the research was conducted in the absence of any commercial or financial relationships that could be construed as a potential conflict of interest.

## Abbreviations

Aβ: beta-amyloid peptide derived from APP
AC: adenylyl cyclase
AD: Alzheimer’s disease
AICD: APP intracellular domain cleavage fragments of APP family proteins
Akt: target of PI3K (also called Protein kinase B)
APP: amyloid precursor protein
APP_695_: predominant isoform of APP in mammalian neurons (695 amino acids)
APLP1 and APLP2: APP-Like-Proteins 1 and 2 (additional APP family members expressed in the mammalian brain)
APPL: APP-Like, the insect ortholog of human APP
BiFC: bimolecular fluorescence complementation
CaMKIV: calcium/calmodulin-dependent protein kinase IV
cAMP: cyclic adenosine monophosphate
CREB: cAMP response element binding protein
CTX: cholera toxin
FAD: familial AD
Gαi: alpha subunit of the heterotrimeric G protein Gi
Gαo: alpha subunit of the heterotrimeric G protein Go
Gβγ: beta/gamma dimeric subunits of heterotrimeric G proteins
GSK3β: glycogen synthase kinase 3 beta
JNK: c-Jun N-terminal kinase
pCREB: phosphorylated CREB
PI3K: phosphatidylinositol-4,5-bisphosphate 3-kinase
PKA: protein kinase A
PS-1: presenilin-1
PS-2: presenilin 2
PTX: pertussis toxin, a selective inhibitor of Gαi/Gαo family of heterotrimeric G proteins
sAPPα: secreted ectodomain fragments of APP generated by α-secretase cleavage. sAPPL, secreted ectodomain fragments of insect APPL (equivalent to sAPPα)
